# Supplementary material for: Genome-Wide Survey and Analysis of Microsatellites in Waterlily, and Potential for Polymorphic Marker Development
Source: Genes (Basel). 2022 Oct 2;13(10):1782. doi: 10.3390/genes13101782 (PMC9601493; doi:10.3390/genes13101782)
Supplement: Supplementary file 1 [file genes-13-01782-s001.zip › Figure S1.pdf]

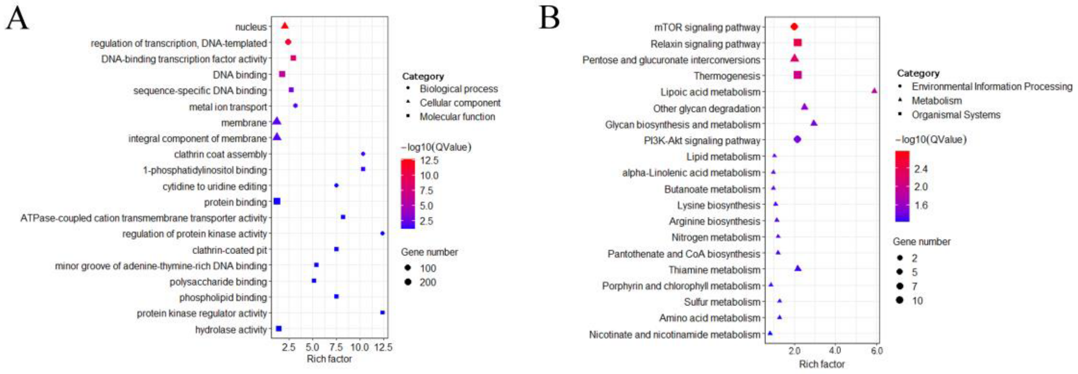

Figure S1. The top 20 significantly enriched analysis of GO terms and KEGG pathways.

GO term enrichment of CDSs with SSR (A). KEGG pathway enrichment of CDSs with SSR (B).
